# Is elastography feasible in torn rotator cuffs before surgery?

**DOI:** 10.1016/j.jseint.2025.10.004

**Published:** 2025-10-30

**Authors:** Mina Shenouda, Nick Bouletos, James Bilbrough, Victor Chen, Ala Hawa, Christyon Hayek, George A.C. Murrell

**Affiliations:** a Orthopaedic Research Institute, St George Hospital Campus, University of New South Wales, Sydney, Australia; b Orthopedics Division, Department of Surgery, Faculty of Medicine, Yarmouk University, Irbid, Jordan

**Keywords:** Shear wave elastography, Rotator cuff, Velocity, Elasticity, Retear, Ultrasound

## Abstract

**Background:**

Shear wave elastography (SWE) is a relatively recent ultrasound imaging technique that uses focused acoustic radiation forces to quantify the elasticity of biological tissues, commonly referred to as ‘elastographic stiffness’. Preliminary data suggest that the elastographic stiffness of a torn supraspinatus tendon may serve as an independent predictor of retear risk following surgical repair. However, the feasibility of obtaining accurate preoperative SWE measurements remains uncertain, as tendon retraction beneath the acromion can limit visualization and reliable assessment.

**Methods:**

This was a prospective cohort study that recruited 60 consecutive patients who had received a diagnosis of supraspinatus tear. SWE imaging was conducted to determine the elastographic stiffness of each patient's torn supraspinatus tendon.

**Results:**

SWE values of the edge of the torn supraspinatus were successfully measured in 59/60 (98%) of cases, resulting in a failure to measure rate of 2%. SWE measurements were not feasible in a single case where the anteroposterior or mediolateral tear lengths were greater than 28 mm. Tendon elastographic stiffness, measured as elasticity (kPa), was inversely correlated with anteroposterior tear length (R^2^ −.51, *P* < .001), mediolateral tear length (R^2^ −.54, *P* < .001), and patient age (R^2^ −.27, *P* < .05).

**Conclusion:**

This study shows that preoperative SWE of the torn supraspinatus tendon is a feasible imaging methodology if the supraspinatus tear is < 30 mm. Patients who had smaller tears and who were younger had greater SWE than older patients with larger tears.

## Background information

Tears of the rotator cuff have been identified as one of the most prevalent musculoskeletal pathologies of the shoulder, accounting for 30%-40% of all reports of shoulder pain.^9^ Supraspinatus tears are often repaired with arthroscopic surgery.^24^ Retear of the supraspinatus tendon has been identified as the most common postoperative complication of arthroscopic repair, with a 2014 retrospective study of 1,000 consecutive repairs and a 2017 prospective study of 206 repairs calculating a retear rate of 17% and 15%, respectively.^2^^,^^15^ Prospective studies of rotator cuff repairs found that the most common mechanism of retear was the tendon pulling through the sutures.^4^^,^^25^ An increased risk of supraspinatus tendon retear has been associated with poor tendon tissue quality. Several studies have identified factors such as fatty infiltration, tendon degeneration, and delamination as contributing to this mechanism of failure.^3^^,^^7^^,^^12^, ^13^, ^14^^,^^16^^,^^22^

Elastographic tendon stiffness is a relatively recent ultrasound-based modality that enables noninvasive assessment of rotator cuff tendon quality and has potential as an independent predictor of retear risk. This measurement is obtained using shear wave elastography (SWE), an imaging technique that applies focused acoustic radiation forces to generate shear waves—transverse displacements that propagate through tissue. The extent of tissue displacement is calculated using a speckle-tracking algorithm to create displacement maps, which are then used to determine shear wave velocity, measured in meters per second (m/s). Shear wave velocity (*c*_s_) and tissue density (ρ) are applied to the equation *G* = ρ*c*,^2^ where *G* represents the shear modulus, a measure of tissue stiffness expressed in kilopascals (kPa).^23^ SWE measurements of tissue stiffness are typically represented in color-coded elastograms, where red indicates stiffer tissues and blue indicates softer tissues.

The earliest applications of SWE to supraspinatus tendons included a study of 3 in-vitro and 3 in-vivo shoulders, which concluded that SWE was a feasible technique to determine the stiffness of rotator cuff tendons.^1^ A 2023 study by Hacket et al^8^ used SWE to evaluate supraspinatus changes postrepair at 1, 6, 12, 24, and 52 weeks in 50 consecutive patients. They found that tendon stiffness at 1 week postoperatively correlated with tendon thickness at 6 weeks. This correlation between tendon stiffness and thickness persisted at the 6-week timepoint, suggesting early postoperative SWE measurements may predict tendon healing and integrity. Therefore, SWE measurements may be a predictor of the structural integrity of the rotator cuff which may assist surgeons in determining whether surgical repair is appropriate for a particular patient.

However, due to medial retraction of the tendon beyond the glenoid, it is not evident whether preoperative SWE measurements of the torn supraspinatus can be reliably taken. A study by Rosskoph et al^20^ found that test-retest reliability varied significantly in patients with varying degrees of retraction. Itoigawa et al^10^ assessed preoperative SWE measurements of torn rotator cuff tendons and noted that the length and elasticity of retracted tendons were not able to be measured due to obstruction by the acromion.

Therefore, the aim of this study was to assess whether SWE measurements of the torn supraspinatus tendon are feasible preoperatively. Using a large prospective database, the study further sought to identify potential cutoff values for anteroposterior (AP) and mediolateral (ML) tear lengths beyond which medial retraction of the tendon edge renders SWE measurement infeasible.

## Methodology

### Study design

A prospective cohort study to determine the feasibility of preoperative SWE measurements of the supraspinatus tendon in patients with rotator cuff tears.

### Ethics

Ethics approval was obtained for this study by the South Eastern Sydney Local Health District Human Research Ethics Committee. Approval number: 2019/ETH05100 — Human Tissue Collection for Orthopaedic Research Institute (ORI; originally HREC 96/55 and 14/130).

### Patient recruitment

Patients were recruited from the cohort of patients diagnosed with a primary rotator cuff tear at our designated clinic. The inclusion criteria consisted of partial or full-thickness tears of the supraspinatus tendon, with SWE measurements attempted preoperatively. The exclusion criteria consisted of rotator cuff tears without supraspinatus involvement, for example, isolated subscapularis tears; concomitant shoulder pathology including glenohumeral arthritis (≥ grade III) or avulsed fracture; and prior surgery in the same shoulder.

During the initial consultation at clinic, a standardized questionnaire was used to gather information on the following parameters: patient age, sex, duration of symptoms, and mechanism of injury (traumatic vs. atraumatic). The size of the tear (ML and AP), as well as the thickness of the tear (partial vs. full), were determined using B-mode ultrasound.

### Shear wave elastography ultrasound assessment

The shear wave elastography ultrasound technique used for the preoperative measurements in this study has been described in 2 2023 papers published by the Orthopaedic Research Institute.^8^^,^^18^ All shear wave elastography ultrasound assessments were conducted by the same musculoskeletal sonographer using the ACUSON Sequoia Ultrasound System (Siemens Medical Solutions, Mountain View, California, USA). All ultrasound measurements were performed by a musculoskeletal sonographer with more than 3 years dedicated experience in shoulder and rotator cuff imaging and established expertise in shear-wave elastography. The SWE measurements were taken while the patient was seated with their elbow bent at 90°, and their hand supinated. The sonographer sat in front of the patient facing them and extended the patient's elbow by 35° of abduction. This position allows for consistent visualization of the middle and posterior edge of the torn tendon, allowing for a reliable method of measuring tendon retraction distance. The ultrasound transducer was placed posteriorly over the supraspinous fossa to visualize the supraspinatus tendon lengthwise. The ultrasound view of the supraspinatus tendon resembled a bird's beak, with the acromion being viewed medially and the greater tuberosity of the humerus being viewed laterally. The transducer was moved anteriorly to display the anterior supraspinatus tendon, extending from the deep fibers of the supraspinatus muscle to the bursal edge of the supraspinatus. The junction of the tendon footprint and the articular cartilage of the humerus provided the clearest view of the supraspinatus tendon, located 2 mm posterior to the biceps.

SWE measurements were taken at 3 points, including 1 point along the tendon and 2 control points, modified from previous protocols.^8^^,^^18^ The 2 control points were the humeral head (high stiffness) and the muscle belly of the deltoid (low stiffness). SWE measurements were obtained at the torn tendon edge. For partial thickness tears, this was the medially retracted margin, and for full thickness tears, the retracted free edge. SWE measurements were taken both as velocity (m/s) and elasticity (kPa) readings, which are equivalent values based on the shear modulus equation.

### Data analysis

IBM Statistical Package for Social Sciences Statistics (IBM Corp., Armonk, NY, USA) software was used to conduct the statistical analysis for this study. The following data were retrieved for each patient in the cohort: age (years), sex (M/F), side of tear (L/R), symptom duration (months), traumatic injury (Y/N), tear thickness (%), AP length (mm), ML length (mm), SWE measurement feasibility (Y/N), supraspinatus tendon velocity (m/s), and elasticity (kPa). Spearman's rank correlation coefficient was used to determine the correlation between the preoperative SWE values and each of the aforementioned factors. The significance value for correlations between these factors was set at *P* < .05.

## Results

### Participant population

**Figure 1:**
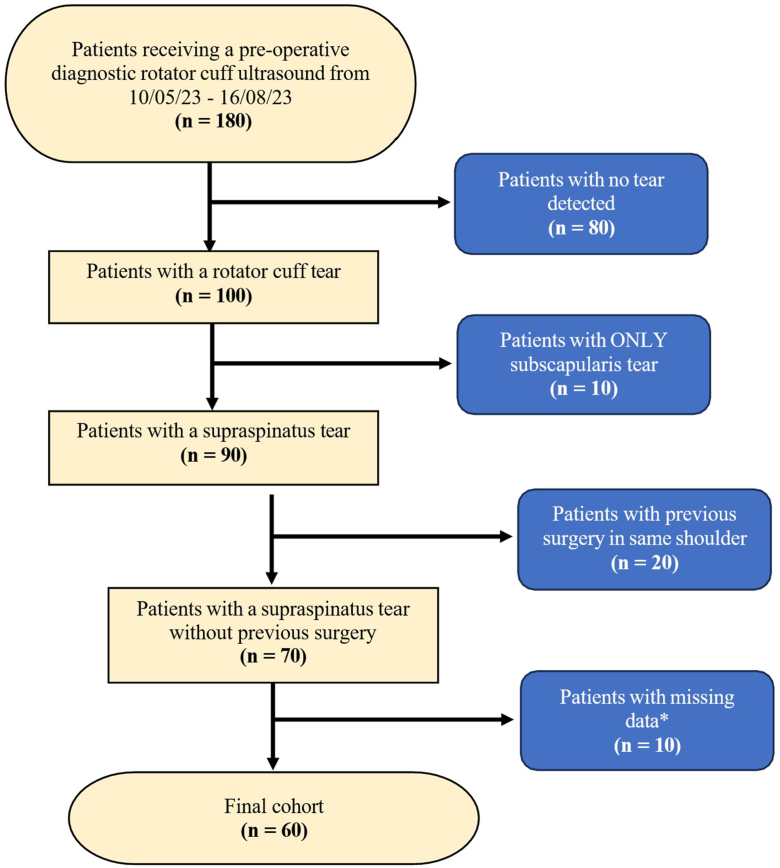
Consort diagram of recruitment and exclusion from initial cohort of 180 patients to final cohort of 60 patients. ∗Missing data included SWE values, AP tear length, ML tear length, tear thickness, age, and symptom duration. *SWE*, shear wave elastography; *AP*, anteroposterior; *ML*, mediolateral.

There were 180 patients who received a preoperative diagnostic rotator cuff ultrasound at the clinic during a 3-month period. Of these 180 patients, 80 were excluded as no tear was detected in their ultrasound. Further 10 patients were excluded because they had an isolated subscapularis tear, with no involvement of the supraspinatus tendon. In addition, 20 patients were excluded due to previous surgery in the same shoulder that was scanned. This left 70 patients diagnosed with a supraspinatus tear, without concomitant pathology or previous surgery. Ten of these patients had insufficient data relating to their tear size and ultrasound results, resulting in a final cohort of 60 patients (Fig. 1).

### Demographic information

In the final cohort of 60 patients, 57% (34) were male, and 43% (26) were female. The mean age of a patient with a supraspinatus tear was 60 years, ranging from 29 to 86 years. Sixty-three percent (38) of the patients had a supraspinatus tear in their right shoulder, while 37% (22) had a tear in their left shoulder. In 73% (44) of cases, patients were able to recall a specific traumatic event that initiated their symptoms, while in the remaining 27% (16) of cases, patients were unable to recall such an event. Finally, average duration of symptoms before ultrasound diagnosis was 33 months, ranging from 0 to 360 months.

The results from B-mode ultrasound showed that 42% (25) of the supraspinatus tears were full thickness tears, while 58% (35) were partial thickness. AP and ML tear length both had a mean of 13 mm, and ranged from 3 mm to 30 mm (AP) and 3 mm to 35 mm (ML).

### Primary outcome: feasibility of preoperative SWE measurement

A total of 60 patients had SWE of their torn supraspinatus tendon and SWE values were successfully measured in 59/60 (98%) of cases. There was 1 case where SWE values not able to be measured, resulting in a failure rate of 2%.

### Secondary outcome: tear length cutoffs for feasible preoperative SWE measurement

There was 1 case among the cohort of 60 patients for which preoperative SWE measurements of the torn supraspinatus tendon were not feasible. This patient was a male aged 73 years, with a nontraumatic right supraspinatus tear and symptom duration of 29 months. Upon ultrasound, the tear was diagnosed as full thickness and irreparable, with an AP length of 30 mm and an ML length of 30 mm.

**Figure 2:**
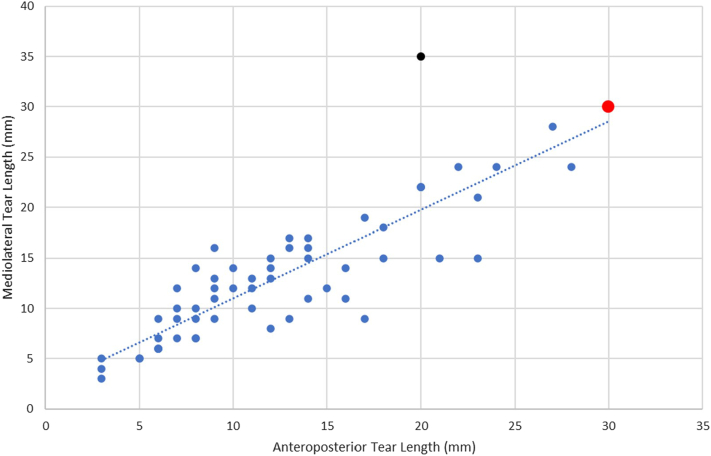
Anteroposterior (AP) and mediolateral (ML) tear lengths of torn supraspinatus tendons as diagnosed by B-mode ultrasound. Spearman's rank correlation coefficient used to correlate AP and ML tear lengths (0.86). ∗∗*P* < .001. Statistically significant if *P* < .05. *Red* data point: case of infeasible shear wave elastography (SWE) (AP 30, ML 30). *Black* data point: Identified outlier (AP 20, ML 35).

Spearman's correlation was conducted between the AP and ML tear lengths of all 60 patients in the cohort. AP and ML tear length were correlated with a Spearman's coefficient of 0.86 (*P* < .001). One data point with AP 20 mm and ML 35 mm was identified as an outlier from the rest of the cohort being well above the fitted trend with a disproportionately large ML length for its AP. Ignoring this outlier, the maximum AP and ML lengths for which preoperative SWE measurements were feasible was estimated to be 28 mm in both dimensions (Fig. 2).

### Secondary outcome: correlations between SWE values, tear length, and patient age

**Figure 3:**
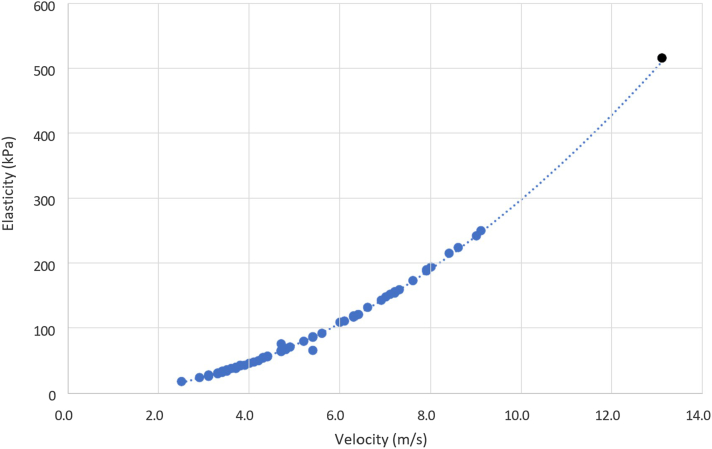
Velocity and elasticity of torn supraspinatus tendons as diagnosed by shear wave elastography ultrasound. Spearman's rank correlation coefficient used to correlate velocity and elasticity (0.99). ∗∗*P* < .001. Statistically significant if *P* < .05. *Black* data point: identified outlier (velocity 13.2, elasticity 519).

Spearman's correlation was conducted between the velocity and elasticity of all 59 successful SWE measurements. Velocity and elasticity were correlated with a Spearman's coefficient of 0.99 (*P* < .001). One data point with velocity 13.2 m/s and elasticity 519 kPa, was identified as an outlier from the rest of the cohort due to its magnitude. As such, this case was removed for the remainder of the data analysis. Ignoring this outlier, the average velocity of the torn supraspinatus tendons was 5.3 m/s with a range from 2.6 m/s to 9.2 m/s. The average elasticity was 95 kPa, with a range from 22 kPa to 254 kPa (Fig. 3).

**Figure 4:**
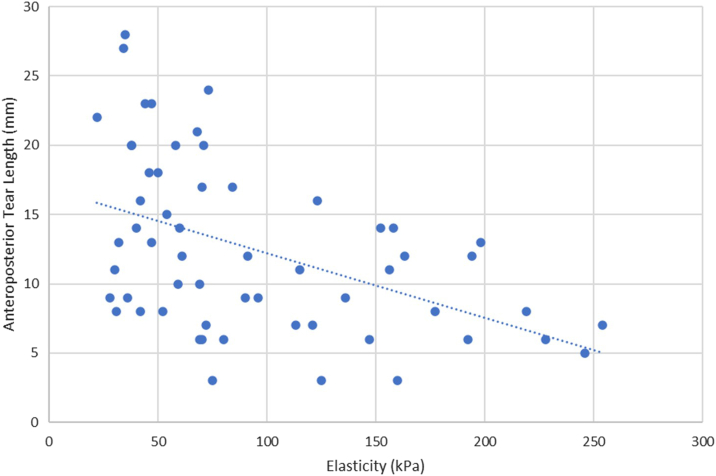
Elasticity and anteroposterior (AP) tear length of torn supraspinatus tendons. Spearman's rank correlation coefficient used to correlate elasticity and AP tear length (R = −0.51). ∗∗*P* < .001. Statistically significant if *P* < .05.

**Figure 5:**
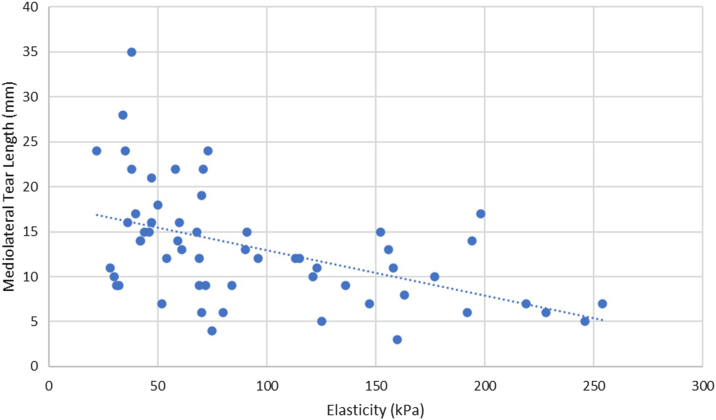
Elasticity and mediolateral (ML) tear length of torn supraspinatus tendons. Spearman's rank correlation coefficient used to correlate elasticity and ML tear length (R = 0.50). ∗∗*P* < .001. Statistically significant if *P* < .05.

**Figure 6:**
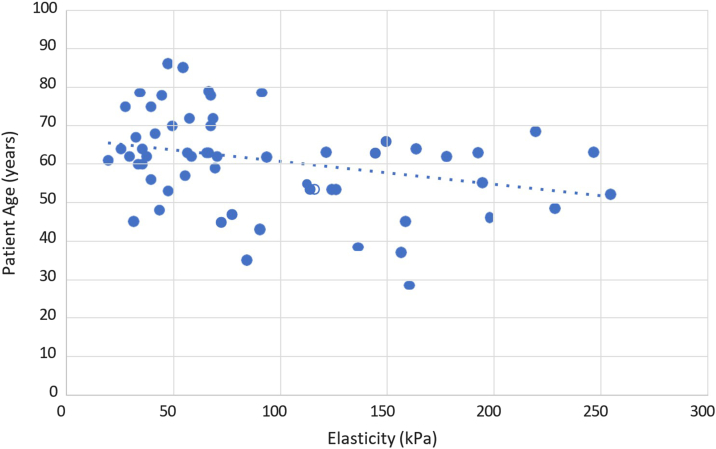
Elasticity and patient age of torn supraspinatus tendons. Spearman's rank correlation coefficient used to correlate elasticity and age (R = 0.27). ∗*P* < .05. Statistically significant if *P* < .05.

**Figure 7:**
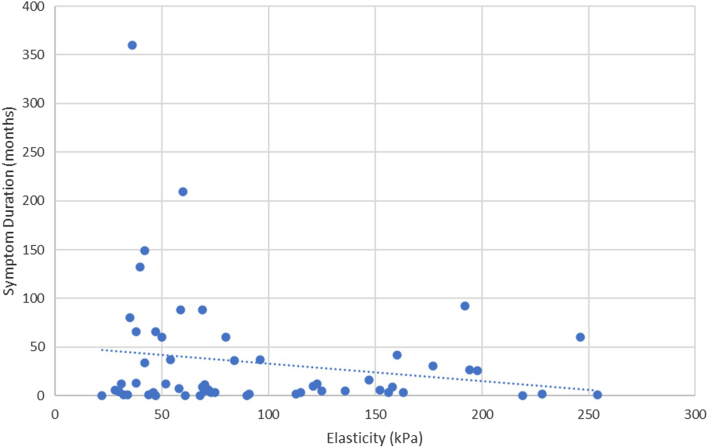
Elasticity and symptom duration of torn supraspinatus tendons. Spearman's rank correlation coefficient used to correlate elasticity and symptom duration (R = 0.01). *P* > .05.

Spearman's correlations were also conducted between the preoperative SWE values of the torn supraspinatus tendons and several secondary factors. Elasticity and AP length were inversely correlated with a Spearman's coefficient of −0.51 (*P* < .001) (Fig. 4). Elasticity and ML length were inversely correlated with a Spearman's coefficient of −0.54 (*P* < .001) (Fig. 5). Elasticity and patient age were inversely correlated with a Spearman's coefficient of −0.27 (*P* < .05) (Fig. 6). No statistically significant correlation was found between elasticity and symptom duration (Fig. 7). Similarly, no statistically significant correlations were found between elasticity and the binary variables to traumatic history and patient sex.

## Discussion

The primary aim of this study was to determine whether it is feasible to take preoperative SWE measurements of the torn supraspinatus tendon. Among the cohort of 60 patients evaluated for tears, the SWE values of their torn supraspinatus tendons were successfully measured in 59/60 (98%) of cases. In only 1 case, SWE values were not able to be measured, resulting in a failure rate of 2%. This result indicates that preoperative SWE measurement of the torn supraspinatus tendon was feasible particularly if the tear was <30 mm.

Previous studies have investigated the feasibility of preoperative SWE measurements for the torn supraspinatus tendon. The study published by Itoigawa et al^11^ in 2020, attempted both preoperative and postoperative SWE measurements in a cohort of 60 patients. The study concluded that preoperative SWE of retracted supraspinatus tendons was not possible because the tendons had “retracted medially”. Itoigawa et al^11^ was contradicted by 2 papers, both published in 2021. The first, published by Nocera et al,^17^ correlated preoperative and postoperative SWE measurements of torn supraspinatus tendons. However, this study included a cohort of only 12 patients. Furthermore, the authors noted “the optimal method or locations to choose specific elasticity measurements was not always clear”. The second paper, published by Ruder et al,^21^ included preoperative SWE measurements in 33 patients. The results of our study support the findings of Nocera et al^17^ and Ruder et al,^21^ confirming that preoperative SWE measurement of the torn supraspinatus tendon is feasible. Our study included a larger cohort of 60 patients, providing a more robust dataset for analysis.

The secondary aim of the study was to determine the cutoff points of AP and ML tear lengths, beyond which preoperative SWE measurements of the torn supraspinatus tendon were no longer feasible. It was found that preoperative SWE measurements were feasible up until an AP length of 28 mm and ML length of 28 mm. However, our data included the removal of 1 outlier with a disproportionate ratio between AP and ML tear length, which could mean that this cutoff may not be applicable to patients with atypical tear dimensions. Furthermore, this cutoff was based only on 1 patient in whom retraction of the torn edge beyond the acromion obstructed SWE measurement.

Previous studies have demonstrated a positive correlation between tear length and the medial retraction of the torn supraspinatus tendon.^5^^,^^6^ In larger tears, the proximal tendon rests beyond the glenoid, between the acromion and spine of scapula. This phenomenon explains the cutoff for preoperative SWE feasibility, as the increased stiffness of bone relative to the supraspinatus tenon prevents elastographic imaging of the underlying soft tissue.^8^

Considering other possible factors, the 1 patient for whom preoperative SWE measurement was not feasible was in the fourth quartile for age and the third quartile for symptom duration. However, this does not suggest a significant relationship between patient age or symptom duration and the feasibility of SWE measurement. Previous studies have linked these factors such as age to tear size. Specifically, a 2017 randomized control trial of 447 patients demonstrated a positive correlation between patient age and tear size.^19^ No such link has been shown between symptom duration and tear size, so it is unlikely that symptom duration had any causal link to the feasibility of preoperative SWE measurement.

The final aim of the study was to investigate the correlation between the preoperative SWE values of the torn supraspinatus tendon and AP and ML tear length. It was found that elasticity and AP length were inversely correlated with a Spearman's coefficient of −0.51 (*P* < .001), and elasticity and ML length were inversely correlated with a Spearman's coefficient of −0.54 (*P* < .001). An additional finding was that elasticity and patient age were inversely correlated with a Spearman's coefficient of −0.27 (*P* < .05). These findings align with a 2023 study by Playford et al^18^ which investigated postoperative SWE values with characteristics of the torn supraspinatus tendon. Playford et al^18^ found that each additional decade of patient age was associated with a 0.5 m/s decrease in supraspinatus tendon shear wave velocity (*P* = .004). In addition, larger tear size was inversely correlated with tendon velocity, with a statistically significant association observed at 6 weeks postoperatively (*P* < .03). However, it should be noted that Playford's study measured SWE values postrotator cuff repair rather than preoperatively.

While our study demonstrated that SWE measurements are feasible preoperatively, it is unknown how preoperative SWE measurements may predict postoperative outcomes. However, preliminary data suggest that SWE values may be useful in predicting outcomes postrotator cuff repair. A 2023 paper, published by Hackett et al,^8^ conducted postoperative SWE measurements for a cohort of 50 patients undergoing rotator cuff repairs. This prospective study found that repaired supraspinatus tendons had increased their SWE measurements by 21% at 6 months postoperatively. Interestingly, patients who had lower SWE measurements at their first scan at 1 week postoperatively were more likely to have thinner tendons at 6 weeks. The results of Playford et al^18^ and Hackett et al^8^ suggest that SWE may serve as a marker of tendon tissue quality and can be used alongside magnetic resonance imaging to potentially estimate tissue integrity and mechanical strength. However, no study to date has investigated the relationship between preoperative SWE values and postoperative outcomes, leaving it unclear whether higher preoperative tendon elasticity can predict improved healing or functional outcomes following surgery. Further research is required to determine whether preoperative elasticity measurements can guide surgeon decision making. In particular, studies should assess their value in predicting repairability, informing the use of graft augmentation, and tailoring rehabilitation protocols.

The main strength of this study was the sample size relative to previous studies on preoperative SWE measurements. The prospective cohort of 60 patients matched the size of Itoigawa et al^11^ and was considerably larger than the 2021 studies published by Nocera et al^17^ (n = 12) and Ruder et al^21^ (n = 33). Furthermore, the cohort consisted of consecutive cases over a period of three months, which were systematically recruited and assessed. In addition, all treatment and imaging were conducted by the same experienced surgeon and musculoskeletal sonographer, who used reliable and previously validated methodologies.^8^^,^^18^

However, this study did have some limitations. While the use of a single surgeon and sonographer increased the internal validity of this study, it limits our external validity. Furthermore, ultrasound examinations were performed by a musculoskeletal sonographer with >3 years clinical experience in shoulder and rotator cuff imaging and hence operator skill may affect the reproducibility of our results. Another limitation of the study was that only a single case resulted in unsuccessful preoperative SWE measurements. This reduced the statistical significance of the findings, particularly in determining the cutoff values for feasibility of preoperative SWE measurement. Furthermore, our patient cohort included both partial thickness and full thickness tears which may confound our findings, particularly the relationship between tear size and tendon elasticity.

## Conclusion

This study found that SWE of the torn supraspinatus tendon is a feasible methodology for preoperative imaging. However, SWE measurements were not feasible beyond AP or ML tear lengths of 30 mm. Tendon elasticity is inversely correlated with AP tear length, ML tear length, and patient age.

## Disclaimers:

Funding: The ultrasound machine was provided to our research group at a reduced price to conduct research.

Conflicts of interest: George Murrell is a paid consultant with Smith and Nephew (Smith and Nephew products may be used with these studies) and is on the Editorial and/or the governing board of the Journal of Shoulder and Elbow Surgery and Shoulder and Elbow (UK). All the other authors, their immediate families, and any research foundation with which they are affiliated have not received any financial payments or other benefits from any commercial entity related to the subject of this article.
